# *OTOF* Gene Therapy: From Breakthroughs to Roadmaps

**DOI:** 10.1002/mco2.70639

**Published:** 2026-03-02

**Authors:** Qiuju Wang, Tobias Moser, Christine Petit

**Affiliations:** ^1^ Department of Audiology and Vestibular Medicine, Institute of Otolaryngology, Senior Department of Otolaryngology Head and Neck Surgery The Sixth Medical Center of Chinese PLA General Hospital Beijing China; ^2^ Institute for Auditory Neuroscience and Inner Ear Lab University Medical Center Göttingen Göttingen Germany; ^3^ Université Paris Cité, Institut Pasteur, AP‐HP, INSERM, CNRS, Fondation Pour l'Audition, Institut de l’Audition, IHU reConnect, F‐75012 Paris, France Collège de France, F‐75005 Paris, France. Université Paris Cité Institut Pasteur AP‐HP INSERM CNRS Fondation Pour l'Audition Institut de l'Audition IHU reConnect Collège de France Paris France

1

In a recent study published in *Nature Medicine*, adeno‐associated virus (AAV)‐mediated *OTOF* gene replacement in autosomal recessive nonsyndromic deafness form 9 (DFNB9) patients restored near‐physiological hearing and markedly improved speech perception [1]. These results move otoferlin therapy from preclinical promise to a realistic, early‐life precision treatment, positioning *OTOF* as a pathfinder for broader genetic cures for hereditary deafness and motivating scalable trial, manufacturing, and regulatory roadmaps.

Remarkably, only 6 years after preclinical proof of concept in mice, *OTOF* gene therapy has achieved transformative clinical milestones [2, 3]. As of now, Chinese teams have conducted the highest number of *OTOF* gene therapy administrations globally, treating a total of 21 patients [4]. By analyzing the publicly available data on *OTOF* gene therapy patients, a comprehensive evaluation was made, discussing successful and less successful cases. This commentary highlights China's leading contributions in *OTOF* clinical trials, delves into the current bottlenecks in genetic therapies for hearing loss, and offers systemic policy recommendations aimed at driving the transition of hereditary hearing loss treatment from exploratory research to routine clinical practice.

Hereditary factors underlie nearly half of global congenital deafness cases, with biallelic *OTOF* mutations causing DFNB9, accounting for a substantial proportion of pediatric auditory neuropathy cases. The *OTOF* locus (NM_001287489.2; chr2:26,680,071‐26,781,624) spans 101,554 bp across 48 Exons, encoding the 1997‐amino‐acid synaptic exocytosis calcium sensor otoferlin [5]. Over 200 pathogenic or likely pathogenic variants have been documented. Epidemiological studies from multiple regions indicate that *OTOF*‐related deafness has been documented worldwide, with a notably high number of reported cases in East Asian populations, including China. The 21 patients treated in China received a gene‐replacement therapy product. Ten patients came from the Anc80L65 single‐arm study (NCT05901480), which enrolled participants aged 1.5–23.9 years, showing the best response in the 5–8 year age group. Several of these participants achieved thresholds close to physiological hearing within 6 months. The other 11 patients were from the AAV1‐hOTOF single‐arm study (ChiCTR2200063181), where children aged 1–11 years showed improvements in pure‐tone thresholds and substantial gain in speech recognition by the 26‐week follow‐up [4]. Both trials reported only mild and transient reversible adverse events, confirming a favorable safety profile and supporting progression to multicenter validation.

Although most recipients show substantial gain of hearing and speech perception within six months, a small subset (three cases to date, Table 1) derived limited benefit from AAV‐*OTOF* therapy. Based on the comprehensive analysis of case phenotypes, preoperative indicators, and perioperative records, the suboptimal therapeutic outcomes may be attributable to the recovery time of the central auditory system, technical and procedural factors, as well as other undefined immunological factors.

**TABLE 1:** Participants with suboptimal outcomes of *OTOF*‐related gene therapy.

No	Sex	Age	Mutations in *OTOF* Allele 1	Mutations in *OTOF* Allele 2	Click‐ABR dB	PTA dB	Injection ear	CI ear	Vector dose administered, vg	Reference
1	Male	1.6	c.2377G>T	c.4225A>T	> 99	112	Left	Right	8.4 × 10^11^ to 11.2 × 10^11^	Qi et al. [1]
2	Male	5.0	c.2215‐1G>C	c.5108delins TCTT	> 95	100	Right	Left	1.5 × 10^12^	Lv et al. [4]
3	Famale	6.0	c.1273C>T	c.3570+2T>C	> 96	120	Right	Left	8.4 × 10^11^	Unpublished

Abbreviations: ABR, auditory brainstem response; CI, cochlear implantation; PTA, pure tone average.

From a global perspective, we identify the following hurdles for a large‐scale rollout of *OTOF* gene therapy and, more broadly, gene therapies for hearing loss. Many patients with hereditary hearing loss, including *OTOF*‐related auditory synaptopathy, are not diagnosed in a timely manner, limiting their access to emerging gene therapies. Further challenges include the low global accessibility to treatment, fragmented GMP production capacity for vectors, lack of unified cross‐border regulatory guidelines, long‐term funding gaps for clinical translation, and non‐harmonized follow‐up evaluation standards around the globe.

In order to promote the sustainable development and accessibility of *OTOF* and genetic therapies for deafness more broadly, the ongoing expert consensus‐building discussions on hereditary hearing loss for gene therapy should be utilized by the community and other stakeholders to develop the relevant standards. Importantly, future work should thoroughly benchmark the outcome of *OTOF* gene therapy to that achieved by cochlear implants, in particular regarding speech development and speech recognition (including in background noise), and assess the quality of life in a comparative manner. Intraindividual comparison will offer a powerful setup to avoid the impact of interindividual outcome variability. Regulators, researchers, and industry should collaborate across six mutually reinforcing domains:

1. *Innovative Exploration*: Facilitate international mutual recognition of AAV production, quality standards, and distribution chains for novel hearing loss gene therapies. Encourage innovation from basic research to clinical application and accelerate translational clinical studies.
2. *Regulatory Coordination and Molecular Diagnosis*: Establish a fast‐track pathway for hearing loss gene therapy by implementing rolling review and mutual recognition and expediting drug evaluation and market access. Simultaneously, promote molecular diagnosis of hereditary deafness to enable precise patient selection and optimize gene therapy outcomes.
3. *Diverse Financing*: Create international joint funds to support projects from basic research to commercialization. Encourage public entities, commercial insurers, and charitable foundations to share the costs of early clinical trials and post‐market real‐world evidence studies.
4. *Data Sharing and Standardization*: Promote widespread genetic testing and integrate genotype‐phenotype data. Develop globally accessible hereditary hearing loss databases, standardized registration platforms, and long‐term follow‐up systems. Harmonize protocols for patient enrollment, surgical procedures, follow‐up assessments, and endpoint evaluations.
5. *Industrial Ecosystem*: Develop regional manufacturing and clinical trial centers in Asia, North America, and Europe. Explore new vector technologies, introduce robotic inner ear drug delivery devices, reduce production and treatment costs, strengthen collaboration across academia, industry, research, and healthcare, and promote coordinated development.
6. *Patient Support*: Enhance patient education and public awareness. Establish comprehensive healthcare systems covering screening, genetic counseling, diagnosis, treatment, and rehabilitation. Advocate for specialized financial support systems to alleviate patient burdens. Strengthen ethical oversight frameworks to ensure fair and equitable global access to these therapies.

Chinese teams were the first to successfully provide proof of concept in humans of the efficacy and safety of a gene therapy product for deafness. Beyond China, the registrational CHORD study of DB‐OTO has shown that a dual AAV1 vector using a hair cell‐specific promoter can also restore acoustic hearing in children with *OTOF*‐related deafness. Nevertheless, efficacy remains limited by genotype‐phenotype variability, the cochlear immune microenvironment, and finite central auditory plasticity. Harmonized regulation, sustained financing, and shared standards could position *OTOF* therapy as the pioneer program, paving the way for treatments targeting other high‐incidence deafness genes (e.g., *GJB2*, *SLC26A4*, and *TMC1*). Through coordinated global collaboration among scientists and clinicians, deafness gene therapy can achieve broad, equitable access for affected individuals worldwide.

## Author Contributions

Q.W. conceptualized the study, acquired funding, supervised the work, and wrote the original draft. T.M. contributed to the conceptualization, provided supervision, drafted the manuscript and editing. C.P. performed the investigation, validated the data and performed the final review. All authors have read and approved the final article.

## Ethics Statement

The authors have nothing to report.

## Conflicts of Interest

Author Tobias Moser is an Editorial Board member of MedComm. Author Tobias Moser was not involved in the journal's review or decisions related to this manuscript. The other authors declared no conflicts of interest.

## Data Availability

The authors have nothing to report.
